# Can women's self‐help groups improve access to information, decision‐making, and agricultural practices? The Indian case

**DOI:** 10.1111/agec.12510

**Published:** 2019-08-19

**Authors:** Kalyani Raghunathan, Samyuktha Kannan, Agnes R. Quisumbing

**Affiliations:** ^1^ Poverty, Health and Nutrition Division International Food Policy Research Institute New Delhi India; ^2^ Markets, Trade and Institutions Division International Food Policy Research Institute New Delhi India; ^3^ Poverty, Health and Nutrition Division International Food Policy Research Institute Washington DC

**Keywords:** agriculture, empowerment, India, information, self‐help groups, J16, O13, O53, Q10, Q18

## Abstract

Effective agricultural extension is key to improving productivity, increasing farmers’ access to information, and promoting more diverse sets of crops and improved methods of cultivation. In India, however, the coverage of agricultural extension workers and the relevance of extension advice is poor. We investigate whether a women's self‐help group (SHG) platform could be an effective way of improving access to information, women's empowerment in agriculture, agricultural practices, and production diversity. We use cross‐sectional data on close to 1,000 women from five states in India and employ nearest‐neighbor matching models to match SHG and non‐SHG women along a range of observed characteristics. We find that participation in an SHG increases women's access to information and their participation in some agricultural decisions, but has limited impact on agricultural practices or outcomes, possibly due to financial constraints, social norms, and women's domestic responsibilities. SHGs need to go beyond provision of information to changing the dynamics around women's participation in agriculture to effectively translate knowledge into practice.

## 1 INTRODUCTION

Agricultural extension systems aim to improve productivity and raise incomes by increasing farmers’ access to information about agricultural practices, prices, and markets, along with the promotion of more diverse sets of crops and improved methods of cultivation. Effective agricultural extension is particularly important in countries where the agricultural sector accounts for the bulk of the country's employment, but where agricultural productivity is low, such as India (Census, 2011; Gillespie, Harris, & Kadiyala, 2012; Planning Commission, 2007). Although recent central government planning exercises in India have emphasized agricultural extension, the coverage of agricultural extension workers, and the relevance of advice provided remains poor (Glendenning, Babu, & Asenso‐Okyere, 2010). Moreover, extension workers typically work with large farmers, who are predominantly male, potentially excluding small, marginal, and women farmers, who comprise a considerable proportion of the farming community but control a small proportion of operational holdings. Women farmers control fewer than 13% of total operational holdings (Agricultural Census, 2010–2010).

Extension directed at women has the potential to increase technical efficiency, improve adoption of technologies that benefit women, and increase production diversity. Interventions aiming to provide women with information may achieve these impacts through empowering women and increasing their decision‐making roles in agriculture. Increasing women's decision‐making role can reduce the wage gap (Hertz et al., 2009) and increase the adoption of drudgery‐reducing technology (Khan, Kishore, & Joshi, 2016). Using nationally representative data from Bangladesh, Seymour (2017) found that a smaller empowerment gap between spouses is associated with higher levels of technical efficiency both on plots that women jointly manage with their spouses, and on those that women do not actively manage. Sraboni, Malapit, Quisumbing, and Ahmed (2014), using the same data set, found increases in women's empowerment in agriculture to be positively associated with energy availability and dietary diversity at the household level. Finally, in rural Nepal, Malapit, Kadiyala, Quisumbing, Cunningham, and Tyagi (2015) found that greater women's empowerment in agriculture mitigates the negative impacts of low production diversity on mothers’ and children's dietary diversity. Extension messages encouraging production diversity may also improve dietary diversity of households who depend on own‐production for food, while effective extension services and collective marketing could help improve market access.

Given the limited reach of government extension services in India and the potential gains from empowering women in agriculture, could another information delivery platform—women's self‐help groups (SHGs)—be effective in providing agricultural information to women farmers, increasing adoption of improved agricultural practices, and improving production diversity and market orientation? Local knowledge, social networks, and participatory training (neglected in traditional extension) are increasingly being recognized as important determinants of technology adoption (Bandiera & Rasul, 2006; Chambers & Pretty, 1993; Foster & Rosenzweig, 1995; Maertens, 2017; Magnan, Spielman, Lybbert, & Gulati, 2015b; Munshi, 2004), and women's groups may be a promising platform to effect change on these fronts. Globally, women's groups have emerged as an important platform for promoting the economic, political, and social empowerment of poor women, and in India, SHGs have become central to many rural development interventions. SHGs are local community groups comprised of 10–20 adult women who meet at regular intervals to deposit small amounts of money into a common pot, from which members can take loans. Under the guidance of the National Rural Livelihoods Mission (NRLM) and other NGOs involved in the formation and strengthening of these groups, SHGs in India are now implementing interventions in agriculture and livelihoods. Professional Assistance for Development Action (PRADAN), one of India's largest NGOs, has worked with women farmers over the last 30 years, and has pioneered efforts in providing agriculture extension for and through women's SHGs in rural India.

In this paper, we evaluate the impact of membership in a PRADAN SHG^1^ on a range of agricultural outcomes. We first describe the pathways through which SHGs can affect agricultural practices, recognizing that women's empowerment affects all these pathways. Using cross‐sectional household survey data from a quasi‐experimental impact evaluation of a multisectoral SHG‐based program being implemented by PRADAN, we provide quantitative measures of the effect of the program on women's access to information on agricultural practices, women's role in agricultural decision making, the use of better agricultural practices, production diversification, and market orientation.

Our paper contributes to several strands of the literature on SHGs and development outcomes. First, we provide some of the first quantitative evidence on the effectiveness of a women's group‐based program in improving access to agriculture‐related information in India. Providing agricultural extension through groups presents an opportunity to overcome the inefficiency of the public extension system, but this modality needs to be tested. Second, we contribute to the growing body of evidence on the impact of these groups on women's empowerment (Karlan, Savonitto, Thuysbaert, & Udry, 2017; also see Brody et al., 2017 for a comprehensive review) by focusing on empowerment in agriculture, measured using the Women's Empowerment in Agriculture Index (WEAI), a recently available standardized measure. Given the frequent exclusion of women from decision‐making in agriculture, globally as well as in India, this is an important area of study. Third, our findings contribute to a relatively unexplored area of research on gender dynamics in agricultural decision‐making and technology adoption. Women can differ in their preferences; for example, in Maharashtra, Khan et al. (2016) find that women tend to prefer labor‐saving technology while men prefer technology that increases profits, possibly because women contribute a large share of unpaid labor in transplanting rice, while men have greater control over how the money is spent. Gender also affects the flow of information about new technologies; for example, in Uttar Pradesh, Magnan, Spielman, Gulati, and Lybbert (2015a) find that women are more likely to have connections with poorer households in their village, which are less likely to adopt agricultural innovations, and hence may not be useful sources of information.

The rest of the paper is organized as follows. Section 2 lays out the conceptual framework and describes the hypothesized pathways to impact. Section 3 describes the context and data, and Section 4 presents the empirical strategy. Section 5 discusses the results, and Section 6 concludes.

## 2 CONCEPTUAL FRAMEWORK

**Figure 1:**
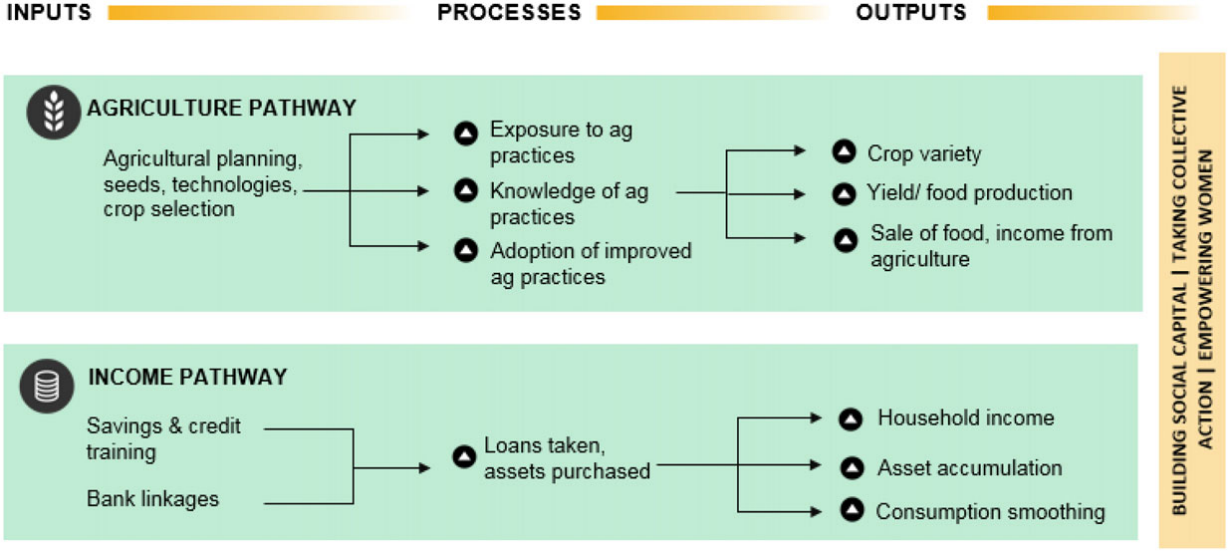
Impact pathways from SHG membership to agricultural outcomes *Source*: Adapted from Kumar et al. (2017). [Color figure can be viewed at http://wileyonlinelibrary.com]

Women's group‐based livelihoods programs may improve agricultural outcomes through multiple pathways (Kumar et al., 2017). Multisectoral interventions such as PRADAN's, typically comprise group formation and capacity building, savings and credit linkages, and livelihoods initiatives, as independent but complementary inputs to improving women's role as farmers. These interventions can affect agricultural outcomes by (a) improving access to inputs, markets, and technical knowledge, or the *agriculture pathway*; (b) increasing access to finance, leading to increased incomes and asset accumulation, or the *income pathway;* and (c) improving women's role in decision‐making on agriculture, or the *empowerment pathway* (Figure 1).

a. Agriculture pathway

Agricultural inputs provided by SHGs include dissemination of information on best practices through a variety of methods, agricultural planning sessions, and increased access to input providers, government schemes, and markets. These livelihoods interventions expose women to, and encourage adoption of, better agricultural practices and improved crop selection. If no other constraints to the adoption of these practices exist, the possible outputs of this pathway are improved crop varieties and increases in yield or food production, market access, and income from the sale of food or cash crops.

b. Income pathway

SHGs typically register with regulators, conduct regular savings activities, open bank accounts, and access credit prior to undertaking income‐generating activities. Participating in the SHG may increase members’ ability to take loans, which is important for poor women who are not deemed creditworthy. Increased access to credit could increase household income and assets and enable the household to smooth its consumption over time. Increased income could also relax budgetary constraints on the household's ability to adopt better agricultural practices, in turn triggering the agricultural pathway.

c. Cross‐cutting pathway: Women's empowerment

Finally, the women's empowerment pathway underlies and interacts with the other impact pathways. SHGs trigger this pathway through building social capital, taking collective action, and empowering women. We expect that the bundling of agriculture and livelihoods programs with the broader women's empowerment agenda will increase women's decision‐making role in farming, which may improve her bargaining power, potentially improving health and nutrition outcomes.

Our paper focuses on all three pathways to impact. We look at the receipt of information on agricultural practices, the use of improved methods of cultivation, production diversity, and market orientation (agriculture pathway). We study household loan taking behavior, access to a bank account, and total household consumption expenditure (income pathway). Finally, we examine women's empowerment and their decision‐making in a range of agriculture‐related activities (women's empowerment pathway).

## 3 CONTEXT AND DATA

### 3.1 Context

Since the 1980s, PRADAN has been working in eight states across India to promote and strengthen SHGs through their agricultural programs. PRADAN has also partnered with state and local governments to manage livelihoods programs under the NRLM framework. As its core activity, PRADAN organizes women's groups, enabling women to acquire financial independence and agency, play a bigger decision‐making role in their household, and empower other women in the community. Groups are initially encouraged to save and lend internally, taking on other tasks as they mature. PRADAN also works extensively with women to generate awareness on gender equality, provide a platform for women to share their personal experiences, and initiate social and political action wherever appropriate.

PRADAN's livelihoods interventions include providing information about improved agricultural practices, demonstrating agricultural techniques, organizing women farmers into producer groups, and providing support to negotiate better prices for their produce. Their extension program is delivered through group meetings and involves field demonstrations of best practices, exposure visits, collective planning for the upcoming agricultural season, entrepreneurial skill development, and linkages to input suppliers and markets. SHG members are encouraged to invite their spouses to join planning meetings for the upcoming agriculture season. In some places, PRADAN has supported women in forming farmer producer organizations that purchase inputs in bulk and make quality inputs available to members at fair prices. These organizations also facilitate aggregating produce to reduce transaction costs and access larger markets. In areas not served by PRADAN, these services are provided by agriculture entrepreneurs who are selected from communities to provide quality inputs, raise high‐quality vegetable seedlings, and provide mechanization and market aggregation support.

### 3.2 Data

We use cross‐sectional data from 2015 to examine the impact of PRADAN's livelihoods programs on several intermediate and final outcomes along our theory of change. Our data are from eight districts across five states in India—Odisha, Madhya Pradesh, Jharkhand, Chhattisgarh, and West Bengal. In each of our eight sample districts, two blocks with PRADAN presence were purposively selected. We assume that all PRADAN SHGs receive the standard inputs of capacity building and monitoring, as well as the livelihoods inputs and focus on improving women's empowerment. From each of the two PRADAN blocks, five villages were randomly selected from the complete list of villages where PRADAN was operational. Finally, 20 ever‐married women between the age of 15 and 49 were randomly selected from each village.^2^ We did not sample based on SHG membership, and since PRADAN saturation in these blocks is less than complete, our sample contains both SHG members and nonmembers. The achieved sample size was 977 women from 80 villages in 16 blocks across eight districts. Of these 977 women, 414 (or about 42%) were SHG members at the time of data collection, and the remaining 563 were nonmembers. Our survey collected data on demographic and socioeconomic characteristics, participation in women's collectives, receipt of agricultural information, cropping practices in the two seasons prior to the survey, and women's empowerment in agriculture, as measured by the WEAI.

The WEAI identifies five domains of empowerment (5DE) (Online Appendix, Table A.1): (a) decision‐making power around agricultural production, (b) access to and decision‐making power over productive resources, (c) control over use of income, (d) leadership in the community, and (e) time allocation (see Alkire et al., 2013, for details). Each domain consists of one to three subindicators. The respondent is identified as being empowered in each subindicator based on predetermined thresholds, or cutoffs. A simple nested weighting structure with equal weights for each domain is used to aggregate scores on these five domains into a subindex called the 5DE score. The 5DE score, therefore, measures the extent to which each individual is empowered, with a higher 5DE score indicating greater empowerment. A comparison of the 5DE scores for a husband–wife pair within the same household can be used to calculate the gender gap in empowerment. This gap is zero in households where the woman is empowered. For households where the woman is *not* empowered, the gender gap provides a measure of the gap in empowerment scores that needs to be closed for the woman to be as empowered as the man. The greater the gender gap, the greater the shortfall in women's empowerment.

In addition to these outcomes, we also use the relative autonomy in production measure, which calculates the extent to which decisions taken are being driven by internal or external motivators. Respondents are presented with three scenarios involving decision‐making—on agricultural production, taking crops to the market, or livestock raising—and with motives for the decisions, and asked which motivation they most identify with. Individuals who are more internally motivated receive a higher score than those who report taking decisions that are externally motivated or forced upon them.

In this paper, we use the individual‐level 5DE scores for the respondent women, the gender gap in empowerment, the score on the relative autonomy measure, and several of the component questions around women's participation in agricultural decision‐making within the household. Although data on the WEAI is available for all respondent women, male household members were interviewed in only slightly more than 60% of the sample, resulting in a smaller sample for the calculation of the gender gap in empowerment scores. In Table A.2 in the Online Appendix, we compare households where the WEAI was administered to both man and woman to those where only the woman responded.

## 4 EMPIRICAL STRATEGY

This paper aims to examine the effect of PRADAN SHG membership on our outcomes of interest; and this section draws from related work on SHGs and other development outcomes (Kumar et al., 2019). We do not compare mean outcomes for SHG members and nonmembers because this approach does not recognize that women who are SHG members are likely to be systematically different from nonmembers. The average difference in an outcome of interest between SHG members and nonmembers, or the difference in unconditional means, would be a biased estimate of impact.

To make unbiased comparisons, we must construct a comparison group from among nonmembers that was similar to the group of SHG members before the SHGs were introduced. Although the preferred approach to constructing the counterfactual is to randomly provide access to the program among similarly eligible individuals, this method was not feasible because SHGs were not randomly introduced across our sample. The absence of “hard” targeting criteria (such as a means test, see Pitt, Khandker, & Cartwright (2006)) precluded the use of Regression Discontinuity Design. Although we explored instrumental variables techniques, the instruments available in our data proved to be weak. Given this, we decided to use matching methods and constructed a comparison group by matching SHG members to nonmembers based on observable respondent, household, and village characteristics. We estimated impacts of SHG membership using nearest neighbor matching (NNM)—a form of covariate matching in which the comparison group sample of nonmembers is selected based on similarity to the SHG member sample in observable characteristics (Abadie & Imbens, 2006, 2008).^3^ We implemented NNM rather than propensity score matching (PSM) because NNM is entirely nonparametric and does not rely on distributional assumptions that underlie probit or logit models used to estimate the propensity scores in PSM; in addition, NNM relies on analytical standard errors rather than bootstrapped standard errors, which are not recommended in the context of matching (Abadie & Imbens, 2008). However, we also used PSM to check the robustness of the results.

Some details and limitations of the matching procedures used deserve attention. It is important to choose variables that are associated both with the probability of being an SHG member and with the outcome of interest (Heckman & Navarro‐Lozano, 2004). However, these variables should be determined before the SHGs were established to ensure that they were not affected by SHG membership itself. Since our data come from a single cross‐section, we do not have data on these observables before the women became members. Therefore, we use variables that are either exogenous or predetermined.

**Figure 2:**
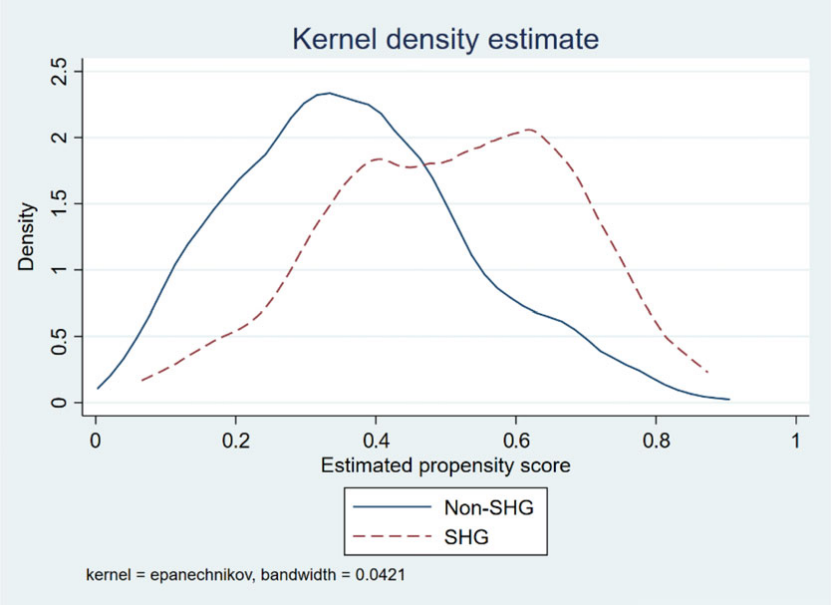
Kernel density of probability of SHG membership for the full sample [Color figure can be viewed at http://wileyonlinelibrary.com]

Table A.3 in the Online Appendix presents the probit model of the probability that the respondent woman belongs to a PRADAN SHG, as a function of a comprehensive list of characteristics (Online Appendix, Table A.4). These include respondent woman characteristics (e.g., age, education, occupation), indicators of her status and time use (e.g., has own disposable income, regularly communicates with own family, fetches water from a distant source, number of hours worked per day), household characteristics (e.g., presence of mother‐in‐law and husband, household size, number of children, caste, size of land owned, whether irrigation is rainfed), and village level characteristics (e.g., population, averages of women's education, size of land owned, wealth, presence of a government primary school, electricity, distance to bank, distance to nearest agricultural wholesale market, and shocks). We also include state and district dummies. These results of the probit show that women's age, education, financial independence, and average land ownership in the village are important correlates of SHG membership. This model is used to compute the propensity score for the matching exercises, to check that the balancing property across the SHG members and nonmembers is satisfied, and to ensure common support of the propensity score between the two groups (Figure 2).

The same list of covariates is then used to match SHG members and nonmembers. Since we use state and district dummies in our matching models as well, we are matching SHG members with nonmembers within the same broad locality. The main limitation of the matching exercise is that it cannot correct for unobservable characteristics that affect both the decision to join an SHG as well as the outcomes of interest. For comparison, we also present the simple ordinary least squares estimates of the relation between SHG membership and the outcomes of interest, using the same list of covariates as in in the matching models.

Our outcomes of interest were chosen to correspond to the various steps along the agriculture, income, and women's empowerment pathways:

Agriculture pathway:

1. Indicators for household receipt of information on field crop selection or rotation, use of improved seeds, line plantation, system of rice intensification (SRI), pest management, soil improvement, irrigation, poultry rearing, livestock rearing, and fishing;
2. Agricultural outcomes: number of crops grown in the summer and winter seasons, the number of food crops, production diversity, and the share of marketed crops. Production diversity is measured using dummies for whether the household planted cereal in summer, cereal in winter, plus one other rotation; or if they planted a cereal in the summer, a pulse in the winter, plus one other rotation.

Income pathway:

1. Whether the respondent woman has a bank account,
2. Whether anyone in the household took a loan in the 12 months preceding the survey,
3. Total household expenditure on food in the last week (in Indian Rupees (INR)^4^),
4. Total household expenditure on durables in the last year (in INR).

Women's empowerment pathway:

1. Composite measures of empowerment from the WEAI—the women's 5DE score and the gender gap in empowerment scores;
2. The number of agricultural domains (out of 10) where the individual has some input in decisions or feels they can make a decision;
3. The sum of the relative autonomy indicators across the three subareas of agricultural production, taking crops to the market, or livestock raising (ranges from −27 to 27);
4. Whether the woman has input into agricultural decisions (food and cash crop farming, livestock, and poultry raising);
5. Whether she feels she can participate to a medium or high degree in decisions on inputs for agricultural production, types of crops to grow, taking crops to the market, and inputs on livestock raising;
6. Whether she takes the decision alone or jointly on adoption of seeds, fertilizer, plant protection measures, and the changing of crops.

The large number of outcome variables makes a *p*‐value correction for multiple hypothesis testing appropriate. Each table (Tables 2, 3, 4, 5, 6) contains both the standard *p*‐value and the corrected *p*‐values that account for the false detection rate. These corrections are based on the suggestions of Benjamini and Hochberg (1995) and Benjamini, Krieger, and Yekutieli (2006), abbreviated as BH1995 and BKY2006, respectively.

## 5 RESULTS

### 5.1 Descriptive statistics

Table 1 presents respondent woman, household, and village characteristics for PRADAN SHG members and nonmembers. On average, SHG women are 34 years old, 2 years older than non‐SHG women. A total of 18.8% of the SHG women have more than primary education, slightly lower than the proportion among non‐SHG women (19.5%). About 48% of SHG women have access to money of their own, and more than half have contact with family members other than those living in their household. On average, SHG women have been members for slightly over 4 years (not shown in table).

**Table 1:** Respondent woman, household, and village characteristics among farming households in PRADAN areas

	SHG women (*N* = 414)	Non‐SHG women (*N* = 563)	
	Mean (SD) or Proportion	Mean (SD) or Proportion	*p*‐Values for tests of difference
**Respondent woman characteristics**			
Age	34.8 (7.8)	32.1 (8.7)	.001
Age‐squared	1,271.5 (544.8)	1,107.1 (580.9)	.002
Has some or all primary education	17.4	14.7	.294
Has more than primary education	18.8	19.5	.849
**Women's status and time use**			
Has money of her own	48.6	43	.131
Talks to own family other than household	55.8	53.3	.553
Fetches water from distant source, summer/winter	41.3	35	.234
Number of work hours per day	4.6 (3.2)	4.3 (3.3)	.172
**Household characteristics**			
Mother‐in‐law is present	20.3	29.1	.001
Husband lives in HH	91.5	89.7	.335
Household size	4.8 (1.9)	4.8 (1.8)	.903
Number of children under 5 in household	0.5 (0.8)	0.6 (0.8)	.098
Household head is from Scheduled Caste (SC)	10.9	11.4	.767
Household head is from Scheduled Tribe (ST)	64	73.2	.131
Household head is from Other Backward Classes (OBC)	18.6	11.2	.059
Amount of farmland owned (in acres)	2.6 (4.4)	2.7 (3)	.689
Rain is the main source of irrigation for crops	89.9	82.4	.086
**Village characteristics**			
Population	834.8 (862.6)	964.9 (1,009.2)	.183
Average education of women	2.3 (1.1)	2.3 (1.2)	.661
Average land owned by a household	2.1 (1.2)	2 (1.1)	.862
Average wealth index in the village	0.6 (0.9)	0.5 (0.9)	.599
Village has at least one government primary school	88.6	88.8	.928
Village has electricity in all areas	75.4	70.3	.150
Distance from the bank (in kilometers)	3.3 (1.2)	3.2 (1.3)	.222
Distance from village to nearest town	21.8 (19.4)	23.2 (18.1)	.513
Livestock loss due to an unexpected event was experienced in the last year	80	84.2	.310
Crop loss due to an unexpected event was experienced in the last year	93	90.4	.462

The average household has slightly fewer than five members and less than one child under the age of 5. More than 60% of the overall sample is Scheduled Tribe (ST); this proportion is higher among non‐SHG women at 73%. Households of SHG members own the same amount of land as the non‐SHG households, about 2.6 acres.

### 5.2 Agricultural pathway

Table 2 presents OLS and NNM estimates of the impact of SHG membership on receipt of information on agricultural practices for PRADAN areas. Because the OLS estimates are likely to be biased, we focus on the matching specification; all effect sizes described in the text refer to the NNM estimates, unless otherwise mentioned.

**Table 2:** Effect of SHG membership on receipt of information: OLS and NNM estimates

	HH received information on:									
	Field crop selection or rotation	Improved seeds	Line plantation	System of Rice intensification	Pest management	Soil improvement	Irrigation	Poultry rearing	Livestock rearing	Fishing
Dependent variable:	(1)	(2)	(3)	(4)	(5)	(6)	(7)	(8)	(9)	(10)
**OLS**										
**Woman belongs to SHG**	0.09**	0.13***	0.07**	0.11***	0.08**	0.06**	0.05	0.02	0.03	0.02
	(0.03)	(0.04)	(0.03)	(0.03)	(0.03)	(0.03)	(0.03)	(0.02)	(0.03)	(0.02)
*Standard p‐value*	.02	.00	.04	.00	.03	.05	.11	.40	.291	.32
*BKY 2006 p‐value* a	.07	.03	.07	.03	.07	.08	.09	.18	.16	.16
*BH 1995 p‐value* b	.08	.02	.08	.02	.08	.09	.15	.40	.35	.35
*R* ^2^	.12	.14	.11	.14	.15	.16	.09	.09	.08	.08
**NNM**										
**Woman belongs to SHG**	0.08**	0.12***	0.07**	0.11***	0.08**	0.07**	0.05*	0.03	0.02	0.03**
	(0.03)	(0.03)	(0.03)	(0.03)	(0.03)	(0.03)	(0.03)	(0.02)	(0.03)	(0.02)
*N_T_*	394	388	392	376	387	385	385	391	389	390
*N_C_*	525	513	526	496	519	515	515	518	516	520
Mean of the control group	0.230	0.205	0.242	0.107	0.211	0.163	0.143	0.094	0.131	0.045

^*^ *p* < 0.1, ^**^ *p* < 0.05, ^***^ *p* < 0.01.

*N_T_*, number of observations in the treatment arm (SHG members); *N_C_*, number of observations in the control arm (non‐SHG members).

a Adjusted *p*‐values calculated based on Benjamini et al. (2006).

b Adjusted *p*‐values calculated based on Benjamini and Hochberg (1995).

The differences in the receipt of information between PRADAN SHG members and nonmembers are large. SHG membership has a statistically significant positive effect on the probability of receiving 8 of 10 types of information, with effect sizes ranging from 3 percentage points (pp) (for fishing) to 12 pp (for improved seeds). These results suggest that SHG members receive more intensive information dissemination than non‐SHG members, with the largest impacts found on improved seeds, SRI, and crop selection or rotation. Increases in information receipt are sizeable relative to the control group mean values presented in the last row of the table. The OLS estimates are very similar to the NNM estimates in all columns and correcting the *p*‐values for multiple hypothesis testing does not significantly alter statistical inference.

Our survey collected data on the source of the agricultural information. Since the proportion of the population receiving information ranged from 5% to 25%, small sample sizes limited our ability to investigate these sources using matching methods. However, tabulating the three most prominent sources of information—government, PRADAN or community organizations, and family/friends—shows that the only statistical difference between SHG and non‐SHG households was in the receipt of information from PRADAN or community organizations. Other responses constituted a small fraction (10% or less). This suggests that the factor driving the differences in information provision is indeed the presence of PRADAN.

Without any measures of knowledge of the agricultural practices, exposure to information on these practices is our closest proxy for actual changes in beneficiary knowledge. We then investigate outcomes further along the agricultural pathway using measures of production diversity and market orientation (Table 3). After accounting for agroecological factors by matching within the same district and controlling for irrigation source, we find a 9 pp impact of SHG membership on the number of crops grown in winter but no impact on the share of crops marketed. However, we find that SHG members are 14 pp more likely to be growing food crops, and 3 (2) pp more likely to be growing cereal–cereal (cereal–pulse) crops in the two seasons along with another rotational crop in between (columns (4) and (5) of Table 3).

**Table 3:** Effect of SHG membership on agricultural outcomes: OLS and NNM estimates

	No. of winter crops	No. of summer crops	No. of food crops	Cereal to cereal, plus rotation	Cereal to pulse, plus rotation	Share of marketed crops
Dependent variable:	(1)	(2)	(3)	(4)	(5)	(6)
**OLS**						
**Woman belongs to SHG**	0.06	0.08	0.13*	0.03	0.01	0.01
	(0.05)	(0.06)	(0.07)	(0.01)	(0.01)	(0.02)
*Standard p‐value*	.29	.20	.09	.11	.34	.61
*BKY 2006 p‐value* a	.51	.51	.51	.51	.51	.65
*BH 1995 p‐value* b	.41	.39	.34	.34	.41	.61
*R* ^2^	.24	.27	.32	.21	.05	.13
**NNM**						
**Woman belongs to SHG**	0.09*	0.04	0.14*	0.03**	0.02**	0.02
	(0.05)	(0.06)	(0.08)	(0.01)	(0.01)	(0.03)
*N_T_*	404	404	404	404	404	404
*N_C_*	546	546	546	546	546	546
Mean of the control group	0.400	1.611	1.941	0.039	0.011	0.237

^*^ *p* < 0.1, ^**^ *p* < 0.05, ^***^ *p* < 0.01.

*N_T_*, number of observations in the treatment arm (SHG members); *N_C_*, number of observations in the control arm (non‐SHG members).

a Adjusted *p*‐values calculated based on Benjamini et al. (2006).

b Adjusted *p*‐values calculated based on Benjamini and Hochberg (1995).

### 5.3 Income pathway

Membership in a PRADAN SHG increases the likelihood of the woman having a bank account by 15 pp, or almost 33% over the baseline mean (Table 4). It also increases by 15 pp the likelihood that someone in the household took a loan in the 12 months preceding the survey, a substantial 70% increase over the baseline mean. We find a positive and significant impact of SHG membership on total household expenditure on durables in the last 1 year of about INR 5,000, an increase of 27% over baseline. We do not, however, see any impact on total household expenditure on food in the week preceding the survey. The OLS estimates are very close to the NNM estimates and correcting the *p*‐values does not alter their statistical significance.

**Table 4:** Effect of SHG membership on outcomes along the income pathway: OLS and NNM estimates

	Respondent woman has a bank account	HH took loan in last 12 months	Total household expenditure on food in last 7 days (INR)	Total household expenditure on durables in last one year (INR)
Dependent variable:	(1)	(2)	(3)	(4)
**OLS**				
**Woman belongs to SHG**	0.15***	0.15***	−9.79	2,478.39
	(0.05)	(0.03)	(19.00)	(2,313.74)
*Standard p‐value*	.01	.00	.61	.30
*BKY 2006 p‐value* a	.01	.00	.44	.25
*BH 1995 p‐value* b	.02	.00	.61	.40
*R* ^2^	.15	.15	.25	.08
**NNM**				
**Woman belongs to SHG**	0.14***	0.15***	−13.20	5,161.986*
	(0.03)	(0.03)	(20.76)	(3,001.31)
*N_T_*	404	404	404	404
*N_C_*	546	546	546	546
Mean of the control group	0.45	0.23	432.48	18,049.26

^*^ *p* < 0.1, ^**^ *p* < 0.05, ^***^ *p* < 0.01.

*N_T_*, number of observations in the treatment arm (SHG members); *N_C_*, number of observations in the control arm (non‐SHG members).

a Adjusted *p*‐values calculated based on Benjamini et al. (2006).

b Adjusted *p*‐values calculated based on Benjamini and Hochberg (1995).

### 5.4 Women's empowerment pathway

Table 5 presents estimates of the impact of SHG membership on measures of women's empowerment. The 5DE and the gender gap in empowerment are set to missing if information on any of the subindicators is missing, so we estimate impacts on a smaller sample, checking that the reduced samples remain well balanced across treatment and control groups (Online Appendix, Figures A.1 and A.2). We find a small but significantly positive (at 10%) impact of SHG membership on the women's 5DE score. SHG membership also results in a large and significant 3 pp (16%) fall in the gender gap in empowerment (column (2)) and a 39 pp (10%) increase in the number of agricultural domains in which the woman has some input into decisions, based on the control means.

**Table 5:** Effect of SHG membership on women's empowerment measures: OLS and NNM estimates

	Women's 5DE score	Gender gap in empowerment scores	Number of agricultural domains individual has some input in decisions or feels can make a decision	Sum of the relative autonomy indicators in the three subareas
Dependent variable:	(1)	(2)	(3)	(4)
**OLS**				
**Woman belongs to SHG**	0.02	−0.03	0.38***	0.11
	(0.02)	(0.03)	(0.10)	(0.52)
*Standard p‐value*	.24	.26	.00	.83
*BKY 2006 p‐value* a	.35	.35	.01	.53
*BH 1995 p‐value* b	.35	.35	.01	.83
*R* ^2^	.15	.18	.16	.13
**NNM**				
**Woman belongs to SHG**	0.03*	−0.03**	0.39**	0.33
	(0.02)	(0.02)	(0.16)	(0.39)
*N_T_*	272	161	377	404
*N_C_*	302	181	495	546
Mean of the control group	0.453	0.184	3.959	−0.117

^*^ *p* < 0.1, ^**^ *p* < 0.05, ^***^ *p* < 0.01.

*N_T_*, number of observations in the treatment arm (SHG members), *N_C_*, number of observations in the control arm (non‐SHG members).

a Adjusted *p*‐values calculated based on Benjamini et al. (2006).

b Adjusted *p*‐values calculated based on Benjamini and Hochberg (1995).

Although these effects are in the expected direction—increasing overall empowerment and reducing the gender gap—it is also useful to look at the WEAI's component indicators to see if there are any offsetting impacts. To investigate this further, we examined women's participation in decision‐making around agriculture‐related actions (Table 6). Sample sizes vary across columns because households do not participate in all activities, but again, the samples remain well‐balanced (Online Appendix, Figures A.3–A.6).

**Table 6:** Effect of SHG membership on women's decision‐making measures: OLS and NNM estimates

	Woman has input into decisions on:				Feels she can participate to medium/high degree in decisions on:				Woman takes decision (alone or jointly) on:			
	Food crop farming	Cash crop farming	Livestock Raising	Poultry raising	Inputs for ag. Prodn	Types of crops to grow	Taking crops to the market	Inputs for livestock raising	Adoption of seeds	Fertilizer	Plant protection	Changing of crops
Dependent variable:	(1)	(2)	(3)	(4)	(5)	(6)	(7)	(8)	(9)	(10)	(11)	(12)
**OLS**												
**Woman belongs to SHG**	−0.02 (0.02)	−0.00 (0.02)	−0.01 (0.02)	−0.06*** (0.02)	0.01 (0.03)	−0.02 (0.03)	−0.01 (0.04)	0.01 (0.04)	0.06* (0.03)	0.06* (0.03)	0.05 (0.03)	0.05* (0.03)
*Standard p‐value*	.38	.99	.49	.00	.67	.52	.70	.86	.06	.07	.11	.10
*BKY 2006 p‐value* a	.81	1.00	.84	.06	.95	.84	.95	1.00	.34	.34	.34	.34
*BH 1995 p‐value* b	.77	.99	.78	.05	.84	.78	.84	.94	.27	.27	.27	.27
*R* ^2^	.11	.14	.13	.18	.16	.16	.13	.16	.23	.25	.26	.25
**NNM**												
**Woman belongs to SHG**	−0.01 (0.02)	−0.04 (0.03)	−0.03* (0.01)	−0.07*** (0.02)	−0.03 (0.04)	−0.03 (0.04)	−0.02 (0.04)	0.00 (0.04)	0.05 (0.03)	0.06** (0.03)	0.06 (0.04)	0.06 (0.03)
*N_T_*	257	132	191	144	397	398	326	354	400	396	351	393
*N_C_*	324	148	241	202	537	536	467	479	536	532	488	530
Mean of the control group	0.954	0.960	0.960	0.985	0.080	0.075	0.053	0.075	0.562	0.534	0.529	0.528

^*^ *p* < 0.1, ^**^ *p* < 0.05, ^***^ *p* < 0.01.

*N_T_*, number of observations in the treatment arm (SHG members); *N_C_*, number of observations in the control arm (non‐SHG members).

a Adjusted *p*‐values calculated based on Benjamini et al. (2006).

b Adjusted *p*‐values calculated based on Benjamini and Hochberg (1995).

Being a member of a PRADAN, SHG has a positive significant 6 pp impact on joint decision‐making in adoption of fertilizer, and the results on adoption of plant protection measures, and decisions around crop rotation are positive, though marginally insignificant (columns (9)–(12)). Counterintuitively, we see a statistically significant decline in women's input into decision‐making around poultry raising and livestock. However, the number of households engaged in these activities is small. While the OLS and NNM results are very similar here as well, correcting the *p*‐values for the false detection rate changes the statistical significance of the OLS estimates substantially, rendering insignificant the association between SHG membership and decision‐making around adoption of seeds and fertilizer use and changing of crops (columns (9)–(10) and (12)).

The control group means reveal that the variation in women's participation in decision‐making depends on the type of decision considered. Women participate almost universally in decisions around food and cash crop farming, livestock raising, and poultry raising, perhaps because these broad decisions are closely linked to household livelihoods. In contrast, women's participation in specific decisions, like what inputs to use for agricultural production, what types of crops to grow, whether to take crops to the market and so on is almost nonexistent, accounting for less than 10% in all cases. It is interesting that the positive results on women's decision‐making do not occur at either extreme, but on those decisions where about half the women are already participating. This suggests that while the SHGs are increasing women's decision‐making in some areas, they have not yet affected those areas perceived as being “traditionally” the men's purview.

### 5.5 Robustness

As a robustness check of the matching algorithm, we reestimated the models with PSM (Online Appendix, Tables A.5–A.9). The PSM results are almost identical to the NNM results in both magnitude and significance, with two exceptions. The first is Table A.6 on agricultural outcomes. Here, the PSM renders insignificant the effect of SHG membership on number of food crops grown, and on the cropping practice of cereal to cereal plus a rotation. The second is Table A.7 on the income pathway, where the PSM results of the effect of SHG membership on expenditure on consumer durables is markedly different from the NNM estimates (though very close to the OLS).

## 6 CONCLUSION

Can SHGs be an effective platform for providing agricultural information to women farmers, improving production diversity, and increasing market orientation? Our answer is a partial yes. We investigated the pathways to impact from membership in SHGs to improved agricultural outcomes, operating through access to information and finance, and through women's empowerment in agriculture. Along the agricultural pathway, we found that women's groups improved access to information, but did not significantly increase the use of improved agricultural practices, number of crops grown, or diversification in cultivation. On the income pathway, SHG membership had large impacts on access to bank accounts, loan taking behavior, and consumer durable expenditure, though not on food‐related expenditure. On the cross‐cutting women's empowerment pathway, women improved their decision‐making power around agriculture, and the gender gap in empowerment within the household decreased.

Some of this lack of impact on production diversity or market orientation could be attributed to imperfect measures. Without plot‐level cropping information, we only have crude measures such as the number of crops grown in each season or the share of crops marketed. These fail to capture changes on the intensive margin, for example, adjustments in the area allocated to each crop, substitution of high‐risk high‐yield varieties for low‐risk low‐yield ones or shifts to crops of greater market value. With this caveat in mind, our results suggest that the effect of SHG membership on desired agricultural outcomes is limited, possibly because of barriers along the pathways to impact.

To make SHGs an effective extension service delivery platform, we need to understand the factors that promote the transmission of information and women's empowerment, as well as those that hinder the translation of knowledge of agricultural practices to actual practice. SHG membership has been shown to increase women's political participation, expand and strengthen their social networks, and increase awareness and utilization of public entitlement schemes, but awareness is not enough. Better general knowledge and increased participation may not result in improved agricultural practices because SHG membership does not improve women's decision‐making related to the *specific* agricultural outcomes of taking crops to the market and decisions around what crops to grow.

Finally, income constraints, limited market access, social norms and traditions, and women's domestic responsibilities may also impede the adoption of improved practices and more diverse cropping patterns. Despite the limited evidence on direct income effects of SHG membership, there is evidence that SHGs empower women economically, and may potentially change the dynamics around agricultural decision‐making and control of resources within the household. Group membership can also change social norms and traditions, particularly those around women's participation in agriculture. NGOs working with SHGs may be able to break the knowledge barrier by providing agriculture extension directly to poor women, and improve women's control over household income, but other barriers that hinder adoption, which may be deeply rooted in social and cultural norms, remain to be addressed. By identifying the gap between knowledge and practice along the SHG impact pathways, our work suggests new areas for future SHG programming and policy research.

## Supporting information


**Figure A.1**: Kernel density of probability of SHG membership for the sample with non‐missing 5DE information (N=574)
**Figure A.2**: Kernel density of probability of SHG membership for the sample with non‐missing gender gap in empowerment information (N=342)
**Figure A.3**: Kernel densities of probability of SHG membership for the sample with non‐missing values on women's input into decisions on food crop farming, cash crop farming, livestock raising and poultry raising.
**Figure A.4**: Kernel densities of probability of SHG membership for the sample with non‐missing values on women being able to participate to some degree in decisions on ag production, types of crops to grow, taking crops to the market, and inputs for livestock raising
**Figure A.5**: Kernel densities of probability of SHG membership for the sample with non‐missing values for women taking decisions on adoption of seeds, fertilizer, plant protection and changing of crops
**Table A.1**: Definitions of the domains of empowerment, and their weights
**Table A.2**: Comparison of households with and without male WEAI respondents
**Table A.3**: Probit model of propensity score estimation
**Table A.4**: List of covariates
**Table A.5**: PSM estimates of the effect of SHG membership on receipt of information
**Table A.6**: PSM estimates of the effect of SHG membership on agricultural outcomes
**Table A.7**: PSM estimates of the effect of SHG membership on outcomes along the income pathway
**Table A.8**: PSM estimates of the effect of SHG membership on women's empowerment measures
**Table A.9**: Effect of SHG membership on women's decision‐making measures

Supporting Information

## Footnotes

1 In this paper, the term “PRADAN area/village” means a village where PRADAN is responsible for the formation of groups either within or outside the NRLM, and other NGOs do not play a role. As a result, women living in PRADAN areas who are SHG members will belong to PRADAN SHGs. For brevity, we will use the term “PRADAN SHGs” to mean all SHGs in PRADAN areas.

2 The data come from the baseline of a larger impact evaluation of a set of nutrition interventions aimed at improving women and children's nutrition, which was why our sample is restricted to ever‐married women of reproductive age.

3 These approaches rely on two assumptions about the data and the model. The first is that, after controlling for all preprogram observable respondent, household, and community characteristics that are correlated with program participation and the outcome variable, nonbeneficiaries have the same average outcome as beneficiaries would have had if they did not receive the program. The second assumption is that for each beneficiary household and for all observable characteristics, a comparison group of nonbeneficiaries with similar observable variables exists.

4 At the time of writing, $1 = INR 70.
