# Supplementary material for: Can women's self‐help groups improve access to information, decision‐making, and agricultural practices? The Indian case
Source: Agric Econ. 2019 Aug 19;50(5):567–80. doi: 10.1111/agec.12510 (PMC6853198; doi:10.1111/agec.12510)
Supplement: Supplementary file 1 — Figure A.1: Kernel density of probability of SHG membership for the sample with non‐missing 5DE information (N=574) Figure A.2: Kernel density of probability of SHG membership for the sample with non‐missing gender gap in empowerment information (N=342) Figure A.3: Kernel densities of probability of SHG membership for the sample with non‐missing values on women's input into decisions on food crop farming, cash crop farming, livestock raising and poultry raising. Figure A.4: Kernel densities of probability of SHG membership for the sample with non‐missing values on women being able to participate to some degree in decisions on ag production, types of crops to grow, taking crops to the market, and inputs for livestock raising Figure A.5: Kernel densities of probability of SHG membership for the sample with non‐missing values for women taking decisions on adoption of seeds, fertilizer, plant protection and changing of crops Table A.1: Definitions of the domains of empowerment, and their weights Table A.2: Comparison of households with and without male WEAI respondents Table A.3: Probit model of propensity score estimation Table A.4: List of covariates Table A.5: PSM estimates of the effect of SHG membership on receipt of information Table A.6: PSM estimates of the effect of SHG membership on agricultural outcomes Table A.7: PSM estimates of the effect of SHG membership on outcomes along the income pathway Table A.8: PSM estimates of the effect of SHG membership on women's empowerment measures Table A.9: Effect of SHG membership on women's decision‐making measures [file AGEC-50-567-s001.docx]

Online appendix


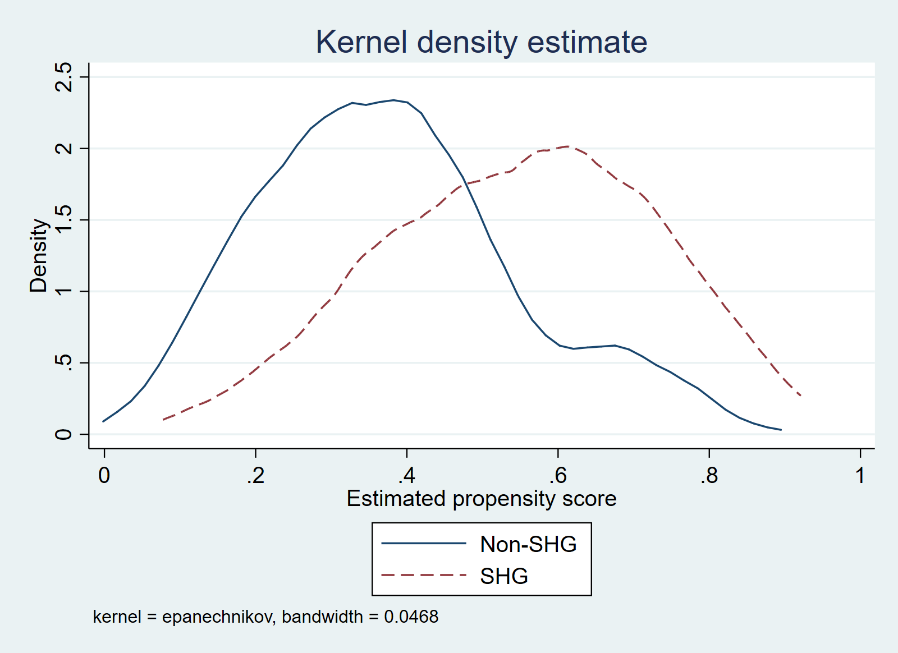


Figure A.1: Kernel density of probability of SHG membership for the sample with non-missing 5DE information (N=574)


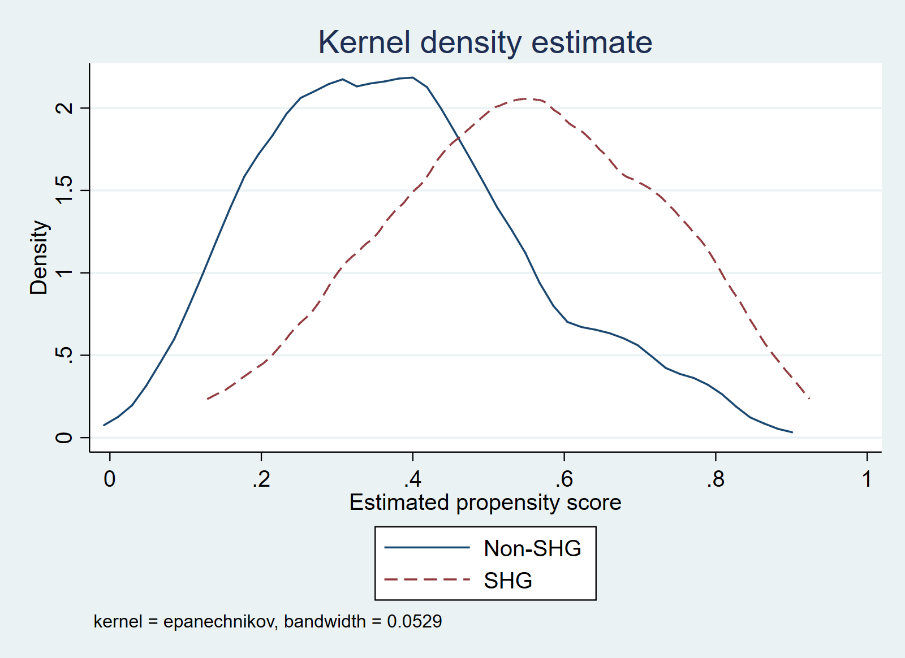


Figure A.2: Kernel density of probability of SHG membership for the sample with non-missing gender gap in empowerment information (N=342)


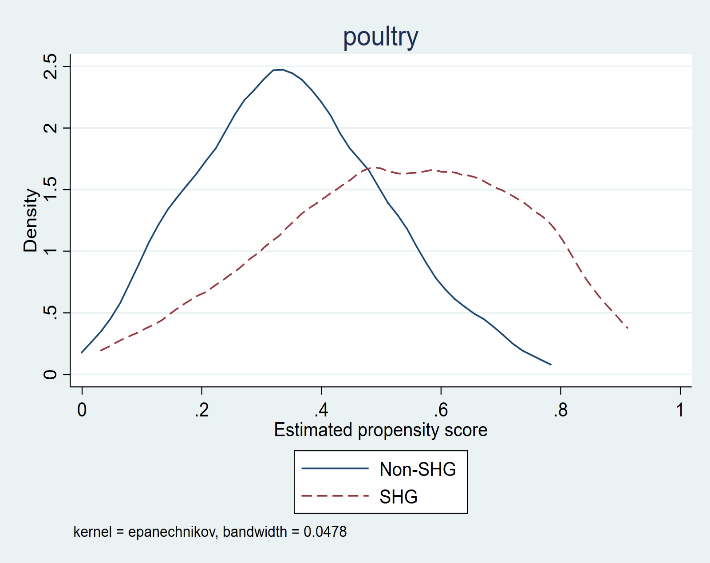

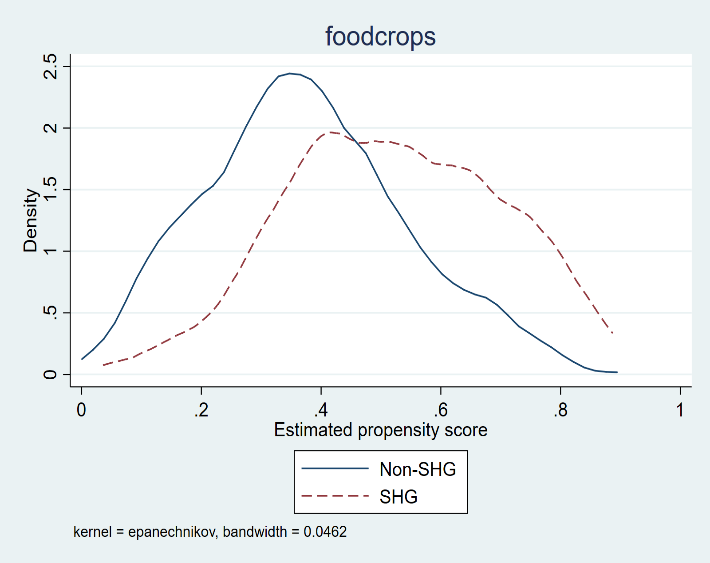

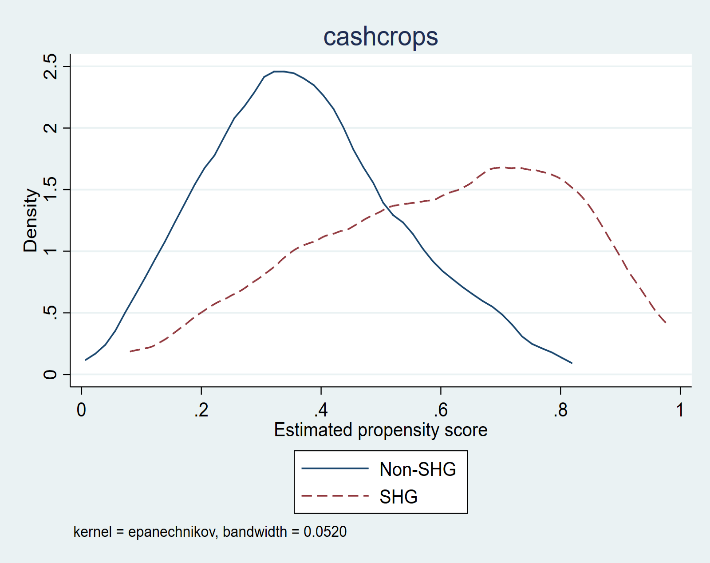

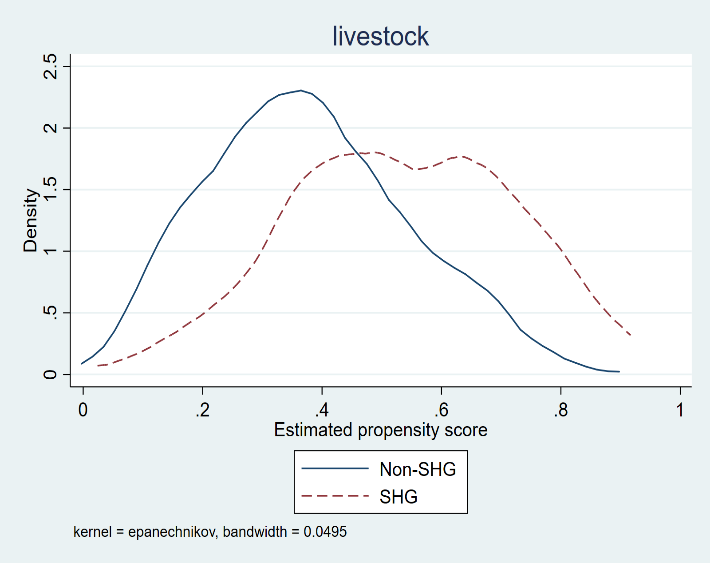


Figure A.3: Kernel densities of probability of SHG membership for the sample with non-missing values on women’s input into decisions on food crop farming, cash crop farming, livestock raising and poultry raising. [columns 1-4 of Table 6]


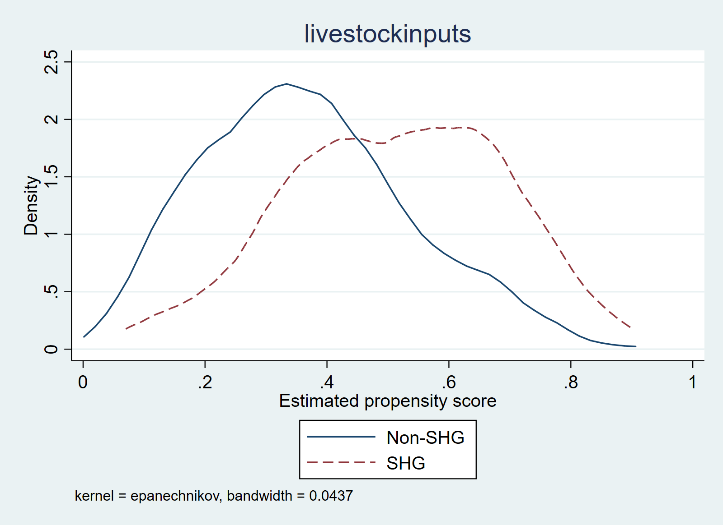

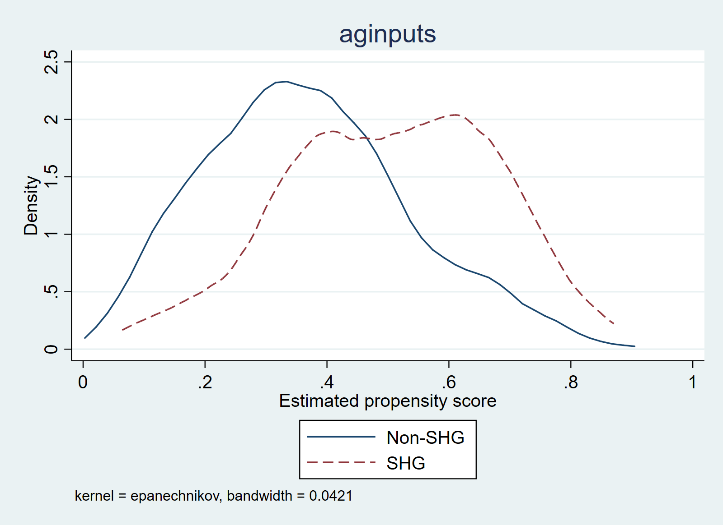

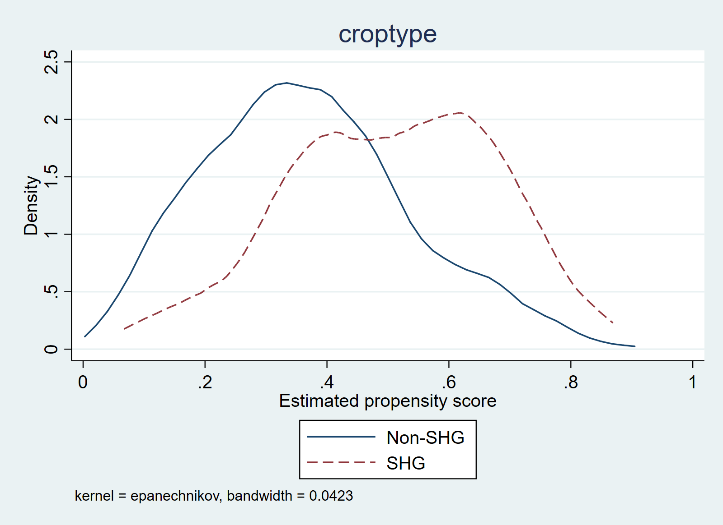

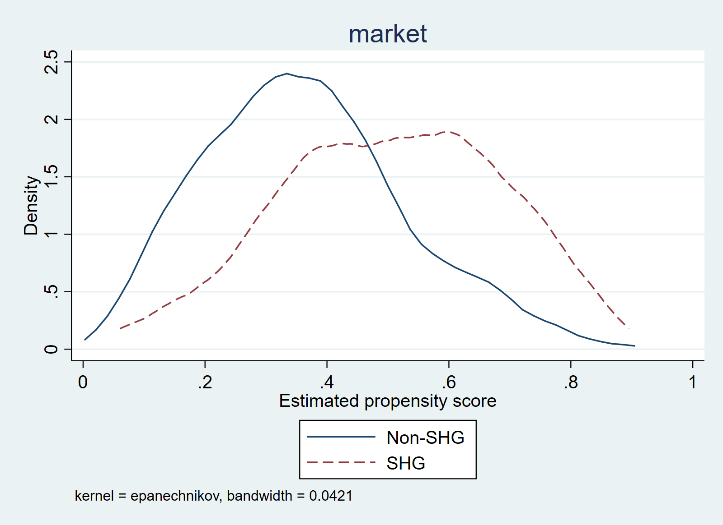


**Figure A.4:** Kernel densities of probability of SHG membership for the sample with non-missing values on women being able to participate to some degree in decisions on ag production, types of crops to grow, taking crops to the market, and inputs for livestock raising [columns 5-8 of Table 6]


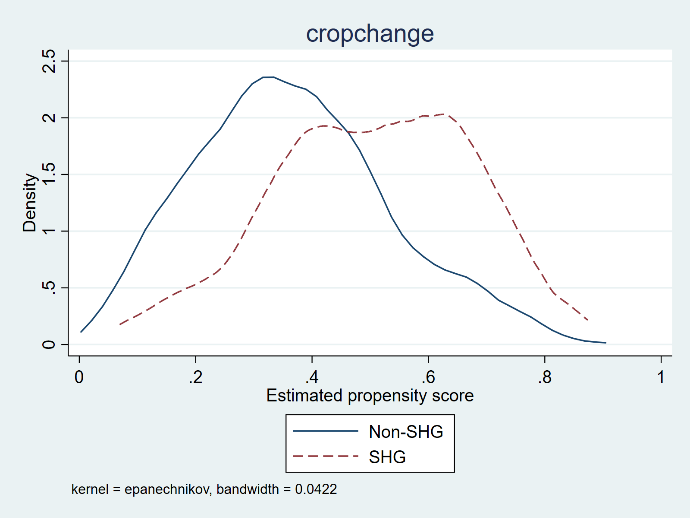

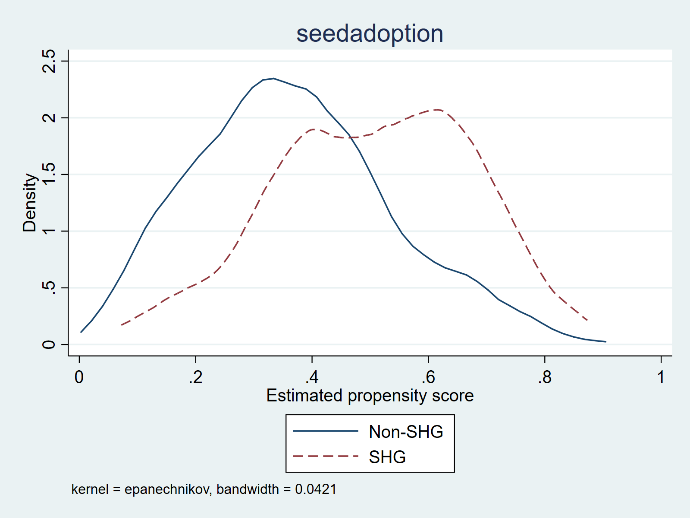

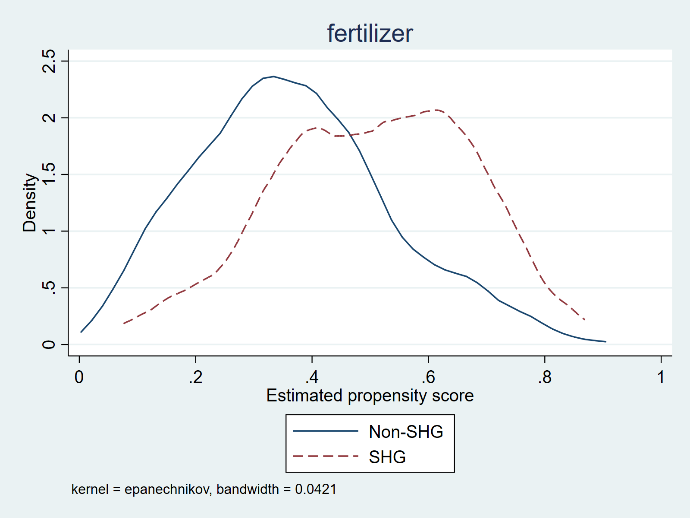

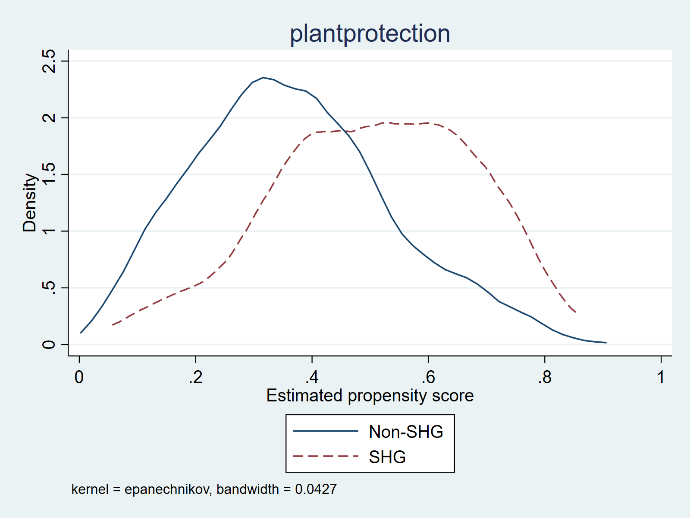


**Figure A.5:** Kernel densities of probability of SHG membership for the sample with non-missing values for women taking decisions on adoption of seeds, fertilizer, plant protection and changing of crops [columns 9-12 of Table 6]

Table A.1: Definitions of the domains of empowerment, and their weights

| **Domain** | **Indicator** | **Definition** | **Weight** |
| --- | --- | --- | --- |
| Production | Input into productive decisions | An individual with at least some input into decisions, makes the decisions or feels they could make the decisions if they wanted, in at least two agricultural domains | 1/10 |
|  | Autonomy in production | An individual who does not strongly agree that her decisions related to at least one of agricultural production, taking crops to the market or livestock raising were externally motivated or coerced | 1/10 |
| Resources | Ownership of assets | An individual who solely or jointly owns at least one large or two small assets | 1/15 |
|  | Right to purchase, sell or transfer agricultural assets | An individual who has at least one joint right to purchase, sell or transfer at least one large or two small household agricultural assets | 1/15 |
|  | Access to and decisions on credit | An individual who has at least one source of formal or informal credit and makes at least one decision solely or jointly for at least one of these types of household credit | 1/15 |
| Income | Control over use of income | An individual with at least some input into decisions about income generated from household agricultural activities or feels she can make decisions in at least one major household income/expenditure domain | 1/5 |
| Leadership | Group membership | An individual who participates in at least one community group | 1/10 |
|  | Speaking in public | An individual who has any degree of comfort when speaking in public in at least one of the three contexts asked about | 1/10 |
| Time | Workload | An individual who works no more than 10·5 h/d including work as an employee; self-employed; in agricultural labour; and domestic work | 1/10 |
|  | Leisure | An individual who does not express any dissatisfaction with the amount of time she has available for leisure activities | 1/10 |

Note: Table reproduced from Malapit et al. (2015) with minor additions.

Table A.2: Comparison of households with and without male WEAI respondents

|  | **Male respondent present (N=1675)** | **No male respondent (N=1069)** | **p-value for test of difference** |
| --- | --- | --- | --- |
| **Household or respondent woman characteristic** | **Mean (SD)/%** | **Mean (SD)/%** |  |
| **Respondent woman characteristics** |  |  |  |
| Respondent woman's age, years | 32.67 (8.43) | 33.23 (8.21) | 0.056 |
| Number of years of education for women | 2.37 (3.63) | 2.18 (3.55) | 0.296 |
| Marital status of woman: married | 98 | 84 | 0 |
| Age at marriage | 17.39 (3.06) | 16.94 (3.04) | 0.001 |
| Age at first pregnancy | 19.16 (3.11) | 18.77 (2.98) | 0.002 |
| Currently member of an SHG | 38 | 39 | 0.754 |
| **Household demographics** |  |  |  |
| Household size | 4.81 (1.8) | 4.41 (1.78) | 0 |
| No. male household members | 2.39 (1.14) | 2.05 (1.18) | 0 |
| No. female household members | 2.42 (1.23) | 2.36 (1.22) | 0.209 |
| Female to male ratio | 1.25 (0.92) | 1.31 (0.95) | 0.175 |
| Dependency ratio | 83 | 98 | 0.001 |
| Religion of household head, Hindu | 86 | 87 | 0.638 |
| Religion of household head, Muslim | 0 | 0 | 0.295 |
| Religion of household head, Christian | 8 | 6 | 0.432 |
| Caste of household head, SC | 13 | 1 | 0.14 |
| Caste of household head, ST | 64 | 71 | 0.03 |
| Caste of household head, OBC | 17 | 15 | 0.294 |
| Highest number of years of schooling in household | 7.18 (3.68) | 6.49 (3.87) | 0.002 |
| Highest number of years of schooling in household, male | 6.33 (4) | 5.76 (3.98) | 0.02 |
| Highest number of years of schooling in household, female | 4.47 (4.04) | 4.2 (4.05) | 0.155 |
| **Household socio-economic characteristics** |  |  |  |
| Household owns home | 97 | 96 | 0.015 |
| Home has electricity | 1.31 (0.46) | 1.37 (0.48) | 0.034 |
| *Type of cooking fuel:* |  |  |  |
| Electricity | 0.24 | 0.47 | 0.274 |
| LPG | 2.93 | 3.37 | 0.615 |
| Kerosene | 0.18 | 0.19 | 0.953 |
| Stone coal | 0.78 | 2.81 | 0.109 |
| Charcoal | 27.1 | 16.65 | 0.07 |
| Wood/straw/leaves | 67.52 | 75.3 | 0.155 |
| Animal dung | 1.19 | 1.12 | 0.901 |
| *Use of improved materials for:* |  |  |  |
| Floor of house | 0.19 | 0.15 | 0.255 |
| Walls of house | 0.25 | 0.24 | 0.49 |
| Roof of house | 0.62 | 0.65 | 0.515 |
| *Ownership of assets, land and animals:* |  |  |  |
| Assets (sum, out of 26) | 5.39 (2.89) | 4.71 (2.87) | 0 |
| Land (in acres) | 2.04 (3.45) | 1.74 (2.62) | 0.024 |
| Large livestock | 2.13 (3.1) | 1.73 (2.69) | 0.007 |
| Small livestock | 1.46 (3.22) | 1.01 (2.55) | 0.001 |
| Poultry | 3.83 (8.19) | 3.01 (11.04) | 0.106 |

Table A.3: Probit model of propensity score estimation

| **Variables** | **Probability of being a PRADAN SHG member** |
| --- | --- |
| Respondent woman's age | 0.20*** |
|  | (0.05) |
| Respondent woman's age squared | -0.00*** |
|  | (0.00) |
| Has some or all primary education | 0.24** |
|  | (0.11) |
| Has more than primary education | 0.28* |
|  | (0.14) |
| Married | 0.42 |
|  | (0.35) |
| Has money of her own | 0.21* |
|  | (0.11) |
| Talks to own family other than household | 0.08 |
|  | (0.07) |
| Fetches water from distant source | 0.09 |
|  | (0.10) |
| Number of work hours per day | 0.01 |
|  | (0.01) |
| Mother-in-law lives in household | -0.12 |
|  | (0.13) |
| Husband lives in the household | -0.05 |
|  | (0.27) |
| Household size | 0.02 |
|  | (0.04) |
| Number of children under 5 in household | -0.01 |
|  | (0.09) |
| Household head is SC | -0.28 |
|  | (0.28) |
| Household head is ST | -0.36 |
|  | (0.25) |
| Household head is OBC | 0.15 |
|  | (0.21) |
| Amount of farmland owned | -0.01 |
|  | (0.01) |
| Rain is the main source of irrigation for crops | 0.38** |
|  | (0.16) |
| Village population | -0.00** |
|  | (0.00) |
| Average education of women in village | -0.05 |
|  | (0.06) |
| Average land owned by a household in the village | 0.13** |
|  | (0.05) |
| Average wealth index in village | 0.07 |
|  | (0.07) |
| Village has at least one government primary school | -0.03 |
|  | (0.11) |
| Village has electricity in all areas | 0.37*** |
|  | (0.10) |
| Distance from the bank | 0.14** |
|  | (0.06) |
| Distance from village to nearest town | -0.01 |
|  | (0.00) |
| Livestock loss due to an unexpected event was experienced in village in the last year | -0.32** |
|  | (0.14) |
| Crop loss due to an unexpected event was experienced in village in the last year | 0.12 |
|  | (0.42) |
| Number of observations | 950 |

Note: Also included are dummies for district. Standard errors in parentheses. ***p<0.01, **p<0.05, *p<0.1.

Table A.4: List of covariates

| **Covariates** | **Definition** |
| --- | --- |
| **Respondent woman** |  |
| Age | Age in years |
| Age-squared | Square of age in years |
| Has some or all primary education | Whether completed class 5/primary school |
| Has more than primary education | Whether completed a class or degree above class 5/primary school |
| Agricultural or non-agricultural day labourer | Whether respondent woman works as an agricultural or non-agricultural day laborer |
| Housewife | Whether respondent is a housewife/homemaker with no additional source of income |
| **Woman’s status and time use** |  |
| Has money of her own | Whether currently possesses or has access to disposable income over which she has full control |
| Talks to own family other than household | Whether communicates with her own family members more than once a month |
| Fetches water from distant source | Whether adult woman in household is responsible for fetching water and the water source is distant from the house. |
| Number of work hours per day | Total number of hours spent at work in a day |
| **Household characteristics** |  |
| Mother-in-law is present | Whether the mother-in-law of the married respondent woman currently resides in the same household |
| Husband lives in the household | Whether the husband of the married respondent woman currently resides in the same household |
| Household size | Number of persons currently residing in the household |
| Number of children under 5 in household^a^ | Number of children less than 5 years currently residing in the same household |
| Household head is SC | Whether the household head belongs to a Scheduled Caste |
| Household head is ST | Whether the household head belongs to a Scheduled Tribe |
| Household head is OBC | Whether the household head belongs to Other Backward Caste |
| Amount of farmland owned | Total farmland owned in acres |
| Rain is the main source of irrigation for crops | Whether the primary source of irrigation for crops cultivated by the household is rainwater |
| Ability to borrow from multiple sources | Whether the household can borrow in cash or kind from more than one source (among NGO, informal lender, formal lender, friends or relatives, group-based microfinance or other women’s groups) if required |
| **Village characteristics**^a^ |  |
| Population | Current population |
| Average education of women | Average of categorical indicator of education level attained by all surveyed women in the village |
| Average land owned by a household | Average land owned in acres by all HHs surveyed in the village |
| Average wealth index | Average of wealth PCA of all respondents in the village |
| Village has at least one government primary school | Whether village has at least one public school. |
| Village has electricity in all areas | Whether village has access to electricity in all areas |
| Distance from the bank | Distance from nearest public or private bank in kilometers |
| Distance to nearest town | Distance from village to nearest town in kilometers |
| Livestock loss due to an unexpected event was experienced in village in the last year | At least one household in the village experienced loss of livestock due to disease or injury etc. |
| Crop loss due to an unexpected event was experienced in village in the last year | At least one household in the village experienced loss of crops due to flooding, drought, disease, animals, theft, etc. |

^a^Refers to village where respondent woman currently resides.

Table A.5: PSM estimates of the effect of SHG membership on receipt of information

|  | HH received information on: | | | | | | | | | |
| --- | --- | --- | --- | --- | --- | --- | --- | --- | --- | --- |
| **Dependent variable:** | **Field crop selection or rotation** | **Improved seeds** | **Line plantation** | **SRI** | **Pest management** | **Soil improvement** | **Irrigation** | **Poultry rearing** | **Livestock rearing** | **Fishing** |
|  | (1) | (2) | (3) | (4) | (5) | (6) | (7) | (8) | (9) | (10) |
| **PSM** | 0.08** | 0.13*** | 0.06** | 0.10*** | 0.06** | 0.06** | 0.04 | 0.02 | 0.02 | 0.02 |
|  | (0.03) | (0.03) | (0.03) | (0.02) | (0.03) | (0.03) | (0.03) | (0.02) | (0.02) | (0.02) |
| N_T_ | 394 | 388 | 392 | 376 | 387 | 385 | 385 | 391 | 389 | 390 |
| N_C_ | 525 | 513 | 526 | 496 | 519 | 515 | 515 | 518 | 516 | 520 |
| Notes:  *p<0.1, ** p<0.05, *p<0.01  N_T_: number of observations in the treatment arm (SHG members), N_C_: number of observations in the control arm (non-SHG members) | | | | | | | | | | |

Table A.6: PSM estimates of the effect of SHG membership on agricultural outcomes

| **Dependent variable:** | **No. of rabi crops** | **No. of kharif crops** | **No. of food crops** | **Cereal to cereal, plus rotation** | **Cereal to pulse, plus rotation** | **Share of marketed crops** |
| --- | --- | --- | --- | --- | --- | --- |
|  | (1) | (2) | (3) | (4) | (5) | (6) |
| PSM | 0.04 | 0.07 | 0.11 | 0.02 | 0.02* | 0.00 |
|  | (0.06) | (0.05) | (0.08) | (0.02) | (0.01) | (0.02) |
| N_T_ | 404 | 404 | 404 | 404 | 404 | 404 |
| N_C_ | 546 | 546 | 546 | 546 | 546 | 546 |
| Notes:  *p<0.1, ** p<0.05, *p<0.01  N_T_: number of observations in the treatment arm (SHG members), N_C_: number of observations in the control arm (non-SHG members) | | | | | | |

**Table A.7:** PSM estimates of the effect of SHG membership on outcomes along the income pathway

| **Dependent variable:** | **Respondent woman has a bank account** | **HH took loan in last 12 months** | **Total household expenditure on food in last 7 days (INR)** | **Total household expenditure on durables in last one year (INR)** |
| --- | --- | --- | --- | --- |
|  | (1) | (2) | (3) | (4) |
| **PSM** | 0.16*** | 0.15*** | 5.53 | 2374.08 |
|  | (0.03) | (0.03) | (20.44) | (2533.50) |
| N_T_ | 404 | 404 | 404 | 404 |
| N_C_ | 546 | 546 | 546 | 546 |

Notes:

*p<0.1, ** p<0.05, *p<0.01

N_T_: number of observations in the treatment arm (SHG members), N_C_: number of observations in the control arm (non-SHG members)

**Table A.8**: PSM estimates of the effect of SHG membership on women’s empowerment measures

| **Dependent variable:** | **Women's 5DE score** | **Gender gap in empowerment scores** | **No. agr. domains individual has some input in decisions or feels can make a decision** | **Sum of the relative autonomy indicators in the three sub-areas** |
| --- | --- | --- | --- | --- |
|  | (1) | (2) | (3) | (4) |
| **PSM** | 0.02* | -0.03** | 0.35** | 0.41 |
|  | (0.01) | (0.02) | (0.14) | (0.37) |
| N_T_ | 272 | 161 | 377 | 404 |
| N_C_ | 302 | 181 | 495 | 546 |

Notes:

*p<0.1, ** p<0.05, *p<0.01

N_T_: number of observations in the treatment arm (SHG members), N_C_: number of observations in the control arm (non-SHG members)

Table A.9: Effect of SHG membership on women’s decision-making measures

|  | **Woman has input into decisions on:** | | | | **Feels she can participate to medium/high degree in decisions on:** | | | | **Woman takes decision (alone or jointly) on:** | | | |
| --- | --- | --- | --- | --- | --- | --- | --- | --- | --- | --- | --- | --- |
| **Dependent variable:** | **Food crop farming** | **Cash crop farming** | **Livestock Raising** | **Poultry raising** | **Inputs for ag. prodn** | **Types of crops to grow** | **Taking crops to the market** | **Inputs for livestock raising** | **Adoption of seeds** | **Fertilizer** | **Plant protection** | **Changing of crops** |
|  | (1) | (2) | (3) | (4) | (5) | (6) | (7) | (8) | (9) | (10) | (11) | (12) |
| **PSM** | -0.02 | -0.02 | -0.02 | -0.07*** | -0.01 | -0.03 | -0.02 | 0.00 | 0.05* | 0.07* | 0.06* | 0.06** |
|  | (0.02) | (0.01) | (0.01) | (0.02) | (0.03) | (0.03) | (0.03) | (0.04) | (0.03) | (0.03) | (0.03) | (0.03) |
| N_T_ | 257 | 132 | 197 | 147 | 397 | 398 | 326 | 354 | 400 | 396 | 351 | 393 |
| N_C_ | 324 | 148 | 247 | 203 | 537 | 536 | 467 | 479 | 536 | 532 | 488 | 530 |
| Notes:  *p<0.1, ** p<0.05, *p<0.01  N_T_: number of observations in the treatment arm (SHG members), N_C_: number of observations in the control arm (non-SHG members) | | | | | | | | | | | | |
